# Health Care Professionals’ Perceptions of Home Telemonitoring in Heart Failure Care: Cross-Sectional Survey

**DOI:** 10.2196/10362

**Published:** 2019-02-06

**Authors:** Ina Thon Aamodt, Edita Lycholip, Jelena Celutkiene, Anna Strömberg, Dan Atar, Ragnhild Sørum Falk, Thomas von Lueder, Ragnhild Hellesø, Tiny Jaarsma, Irene Lie

**Affiliations:** 1 Centre for Patient-Centered Heart and Lung Research Department of Cardiothoracic Surgery Oslo University Hospital Ullevål Oslo Norway; 2 Department of Nursing Science Institute of Health and Society University of Oslo Oslo Norway; 3 Clinic of Cardiac and Vascular Diseases Institute of Clinical Medicine, Faculty of Medicine Vilnius University Vilnius Lithuania; 4 Division of Nursing, Department of Medical and Health Sciences Linkoping University Linkoping Sweden; 5 Department of Cardiology B, Oslo University Hospital Oslo Norway; 6 Institute of Clinical Sciences University of Oslo Oslo Norway; 7 Research Support Services Oslo Centre for Biostatistics and Epidemiology Oslo University Hospital Oslo Norway; 8 Division of Nursing Department of Social and Welfare Studies Linkoping University Norrkoping Sweden

**Keywords:** nurses, physicians, perception, telemedicine, heart failure, self-care

## Abstract

**Background:**

Noninvasive telemonitoring (TM) can be used in heart failure (HF) patients to perform early detection of decompensation at home, prevent unnecessary health care utilization, and decrease health care costs. However, the evidence is not sufficient to be part of HF guidelines for follow-up care, and we have no knowledge of how TM is used in the Nordic Baltic region.

**Objective:**

The aim of this study was to describe health care professionals’ (HCPs) perception of and presumed experience with noninvasive TM in daily HF patient care, perspectives of the relevance of and reasons for applying noninvasive TM, and barriers to the use of noninvasive TM.

**Methods:**

A cross-sectional survey was performed between September and December 2016 in Norway and Lithuania with physicians and nurses treating HF patients at either a hospital ward or an outpatient clinic. A total of 784 questionnaires were sent nationwide by postal mail to 107 hospitals. The questionnaire consisted of 43 items with close- and open-ended questions. In Norway, the response rate was 68.7% (226/329), with 57 of 60 hospitals participating, whereas the response rate was 68.1% (310/455) in Lithuania, with 41 of 47 hospitals participating. Responses to the closed questions were analyzed using descriptive statistics, and the open-ended questions were analyzed using summative content analysis.

**Results:**

This study showed that noninvasive TM is not part of the current daily clinical practice in Norway or Lithuania. A minority of HCPs responded to be familiar with noninvasive TM in HF care in Norway (48/226, 21.2%) and Lithuania (64/310, 20.6%). Approximately half of the HCPs in both countries perceived noninvasive TM to be relevant in follow-up of HF patients in Norway (131/226, 58.0%) and Lithuania (172/310, 55.5%). For physicians in both countries and nurses in Norway, the 3 most mentioned reasons for introducing noninvasive TM were to improve self-care, to reduce hospitalizations, and to provide high-quality care, whereas the Lithuanian nurses described ability to treat more patients and to reduce their workload as reasons for introducing noninvasive TM. The main barriers to implement noninvasive TM were lack of funding from health care authorities or the Territorial Patient Fund. Moreover, HCPs perceive that HF patients themselves could represent barriers because of their physical or mental condition in addition to a lack of internet access.

**Conclusions:**

HCPs in Norway and Lithuania are currently nonusers of TM in daily HF care. However, they perceive a future with TM to improve the quality of care for HF patients. Financial barriers and HF patients’ condition may have an impact on the use of TM, whereas sufficient funding from health care authorities and improved knowledge may encourage the more widespread use of TM in the Nordic Baltic region and beyond.

## Introduction

### Background

Worldwide health care undergoes great changes where caring for chronically ill patients such as heart failure (HF) is expected to take place to a great extent in their homes [1,2]. To be able to perform safe and high-quality care, the use of telemonitoring (TM) is suggested to be widely implemented [3]. The Institute of Medicine defines telemonitoring as “monitoring patient status at a distance by the use of audio, video, and other telecommunications and electronic information processing technologies” [4]. Noninvasive TM detects decompensation at home, prevents unnecessary health care utilization, and decreases health care costs [5,6]. This is important because HF affects 26 million people worldwide, with a rapidly escalating prevalence in Europe and the United States because of an aging population as well as the improved treatment and survival of patients with cardiac disease [7-9]. HF is a complex, progressive clinical syndrome characterized by high mortality, high morbidity with high readmission rates (25% in Europe [7] and 27% in the United States [10]), and affecting quality of life [11]. At present, TM in HF care has tested numerous devices and systems [12]; however, implementation of noninvasive TM in daily HF clinical practice is scarce. Moreover, the evidence for noninvasive TM is not found sufficient to be part of follow-up care recommended by the European Society of Cardiology (ESC) guidelines or the American Heart Association guidelines [7,8]. Results from studies are not consistent regarding outcomes of TM. Some studies have shown that TM may improve survival, reduce HF-related hospitalizations, and improve quality of life compared with usual care or low access to care [2,13,14]; other studies have shown no improvements [15-17]. Furthermore, noninvasive TM studies have methodological weaknesses such as insufficiencies regarding large sample size, homogenous protocols, robust designs, a clear definition of noninvasive TM, and show a diversity of outcomes [5,18,19]. A program where patients monitor their condition at home and transmit information to external centers is not widely established. According to the third global survey on eHealth performed by The World Health Organization, only 22% of the responding countries use TM [3]. Furthermore, in countries with advanced information technology, it is identified that health care professionals (HCPs) are not familiar with using noninvasive TM in their daily follow-up care of HF patients [20]. According to HCPs’ factors of success in using TM, the possibilities include improved diagnostics, improved communication with the patient, and improved support of patient centered care, whereas barriers include workflow and staff turnover [21]. Furthermore, HCPs raise questions on how to assess available technology, the value of using technology, and the evidence of effectiveness and knowledge [22]. TM has been reported to increase the workload for HCPs and the use of health care resources, which may be the reason it has not been widely implemented in clinical practice [23,24].

### Objectives

Although the governments of Norway and Lithuania have approved the use of remote medical services in a new electronic health (eHealth) strategy, little is known on the actual use of TM in daily HF clinical care in the Nordic Baltic region [25,26]. Therefore, this study aimed to describe HCPs’ (1) perceptions of and presumed experience with noninvasive TM in HF care, (2) perspectives of the relevance of and reasons for applying noninvasive TM, and (3) barriers to the use of noninvasive TM.

## Methods

### Study Design and Definition

We conducted a cross-sectional nationwide survey of noninvasive HF TM in Norway and Lithuania.

TM in the survey referred to noninvasive TM, which is implemented via internet-based personal devices monitoring body weight, blood pressure, heart rate, dyspnea, and other signs and symptoms that would reflect the actual volume status of HF patients. Patients use the devices in their home environment, and the generated data are transferred to health care providers over the internet. The information obtained is presumed to reflect the actual condition of HF patients (contrasted with patients’ self-reports) and includes feedback to patients about their condition. Examples of TM implemented via only telephone, telephone support, telephone follow-up, or implantable devices or pacemakers were not considered in the survey. This definition was presented to the participants on the front page of the questionnaire (Multimedia Appendix 1).

### Study Setting and Participants

Norway and Lithuania have 5 and 3 million inhabitants, respectively, and both countries are located in the northern part of Europe. Norway is a high-income country in which the health care system is funded by public sources [27]. Lithuania has undergone political and economic changes with a mixed health system predominantly funded by the National Health Insurance Fund (61% of funding in 2010) and supplemented by a substantial state contribution [28].

The inclusion criteria were nurses and physicians (ie, HCPs) currently working with HF patients in a hospital ward or in an outpatient clinic in Norway or in Lithuania. HCPs were recruited from a list of all potential public and private hospitals caring for HF patients in Norway (N=60) and Lithuania (N=47). The former list was extracted from the Norwegian Heart Failure Registry and the latter from the National Insurance Funds list of hospitals in Lithuania. In addition to telephone follow-up, we contacted the head of each hospital ward and outpatient clinic by post for approval.

### Data Collection and Questionnaire

Data were collected from September to December 2016 using a questionnaire mailed by post. It was sent to 784 physicians and nurses at 107 public and private hospitals providing HF care in Norway or Lithuania. A study researcher in each country (ITA and EL) made 1 phone call to remind the contacted individuals at each site (ward or outpatient clinic) about completing and returning the questionnaire.

#### Questionnaire

A 43-item questionnaire (Multimedia Appendix 1) for assessing HCPs’ perceptions on the use of TM and potential use of TM was developed. It was based on the initial versions of a survey used in the Netherlands [29] and with HCPs in Japan and Sweden [20]. The questionnaire used for this study in Norway and Lithuania included open- and closed-ended response options, with additional questions on HCPs’ characteristics, their perceptions of TM, and potential experiences with TM. Language and cultural adjustments were made from the preparation stage to the final report according to the Principles for Good Practice for the Translation and Cultural Adaption Process for Patient-Reported Outcome Measures [30]. Face validity (measuring target construction) and content validity (relevance, comprehensiveness, and balance) assessments were conducted [31] by 5 cardiologists and 10 nurses with expertise in daily clinical HF care in Norway or Lithuania. These professionals deemed that the questionnaire measured the intended HCPs’ perceptions of TM and potential experiences with TM in HF care.

The questionnaire contained 3 main parts: (1) general questions about the participants and their experiences with information and communications technology (ICT) in general and TM in particular, (2) questions for users of TM, and (3) questions for nonusers of TM. In part 1 of the questionnaire, data on HCPs’ characteristics were collected, with additional questions on education and competency in ICT. The participants with presumed experience and familiarity with TM were asked to respond yes or no to these questions. In parts 2 and 3 of the questionnaire, potential TM user and nonuser participants answered similarly detailed questions about TM. HCPs were asked what they considered to be good ways for performing follow-up of stable HF patients (eg, outpatient clinic, using noninvasive TM, and home visits by a nurse), and a follow-up by a general practitioner (GP) was added to the statement list.

In line with the recommendations by previous users of the survey regarding the low response rate by users with experience with TM [20], we added items to describe HCPs’ perceptions of noninvasive TM: future purpose, criteria, relevance, and feasibility of TM in daily HF care in the participants’ country presented as categories to be marked by the participant. HCPs were asked to rate how important 10 statements were for introducing TM in the care of HF patients (eg, offering higher-quality care and reducing costs) by importance level on a 10-point scale, ranging from *0* for not important to *10* for very important. HCPs responded to how long they considered appropriate for using TM by responding to statements of duration. Finally, HCPs’ perceptions of funding responsibility and situations they thought inappropriate for TM were reported by additional open-ended questions to get an understanding of barriers to the implementation of noninvasive TM.

### Data Analysis

#### Statistical Analysis

All data were analyzed using the Statistical Package for Social Sciences, version 24 (IBM Corp Released 2016 IBM SPSS Statistics for Windows, Version 24.0. Armonk, NY: IBM Corp). Descriptive analysis was presented as means with SDs or SE of the mean and median with interquartile range for continuous variables. Categorical variables are presented with numbers and percentages.

#### Summative Analysis of the Open-Ended Questions

Answers to the open-ended questions were translated from Norwegian and Lithuanian into English by 2 independent researchers (ITA and EL). A total of 44 double-spaced pages of transcripts were produced. First, responses were formulated like statements. Transcripts from both countries were thoroughly read to gain an understanding of the words or statements. In total, 4 authors (ITA, EL, JC, and IL) independently reread the responses to the open-ended questions before reaching consensus on categories and subcategories. Following Hsieh and Shannon, a summative content analysis was performed with numbers and percentages for the subcategories [32].

### Ethical Consideration

All participants signed a written informed consent form before participation. The data protection officer at Oslo University Hospital, Oslo, Norway and University Hospital Santariskiu Klinikos, Vilnius, Lithuania granted consent to perform the study. The study was conducted in compliance with the principles of the Declaration of Helsinki.

## Results

### Participants

The overall response rate of the nurses and physicians was 68.4% (536/784) from 98 of the 107 contacted hospitals in Norway (57/60) and Lithuania (41/47). Characteristics and ICT competency of the participants are summarized in Table 1.

**Table 1:** Characteristics and information and communications technology competency of physicians and nurses in Norway (N=226) and Lithuania (N=310).

Characteristics		Norway		Lithuania	
		Physicians (n=63)	Nurses (n=163)	Physicians (n=137)	Nurses (n=173)
**Gender, n (%)**					
	Female	16 (25)	151 (93.2)	107 (78.1)	171 (98.8)
	Male	47 (75)	11 (6.8)	30 (21.9)	2 (1.2)
Age (range: 23-76 years), mean (SD)		48 (11)	45 (11)	51 (12)	46 (9)
**Education degree**					
	PhD, n (%)	19 (30)	—^a^	37 (27.0)	—
	Master, n (%)	12 (19)	13 (8.0)	100 (73.0)	14 (8.1)
	Bachelor, n (%)	—	150 (92.0)	—	64 (37.0)
	Other type of degree, n (%)	32 (51)	—	—	95 (54.9)
	Post graduate experience (years), median (IQR^b^)	19 (12-28)	16 (9-25)	28 (15-35)	26 (20-32)
**Work time, n (%)**					
	Full-time	32 (51)	59 (36.2)	44 (32.1)	115 (66.5)
	Part-time days/week	19 (30)	64 (39.3)	38 (27.7)	16 (9.2)
	Part-time hours/week	10 (16)	30 (18.4)	54 (39.4)	35 (20.2)
	Unreported or missing	2 (3)	10 (6.1)	1 (0.7)	7 (4.0)
**Hospital level, n (%)**					
	University	11 (18)	25 (15.3)	47 (34)	88 (51.0)
	Second or third	48 (76)	128 (78.5)	82 (60)	80 (46.2)
	Private	4 (8)	7 (4.3)	8 (6)	3 (1.7)
	Unreported or missing	0 (0)	3 (1.8)	0 (0)	2 (1.0)
**ICT^c^** **competency**					
	Computer experience (years), median (IQR)	25 (20-30)	20 (16-25)	15 (10-20)	12 (10-17)
	Operating system, n (%)	60 (95)	158 (96.9)	126 (92.0)	149 (86.1)
	Programs, n (%)	61 (97)	153 (93.9)	109 (79.6)	124 (71.7)
	Programming language, n (%)	11 (18)	43 (26)	18 (13)	22 (13)
	Email, n (%)	63 (100)	162 (99.4)	137 (100)	163 (94.2)
	Email mobile phone, n (%)	56 (89)	154 (94.5)	108 (78.8)	118 (68.2)
	Internet, n (%)	63 (100)	162 (99.4)	137 (100)	167 (96.5)

^a^Participants did not have the degree in question.

^b^IQR: interquartile range.

^c^ICT: information and communications technology.

Among the responders, 28% and 44% worked as physicians in Norway and Lithuania, respectively. The majority of HCPs in Lithuania were female (90%), whereas in Norway, the majority of the physicians were males (75%). In Lithuania, about half of the nurses worked at a university hospital (88/173), whereas physicians more often worked in a second- or third-level hospital (82/137). In Norway, most HCPs worked at a second- or third-level hospital.

A total of 51% (32/63) of Norwegian physicians worked full-time with HF patients and one-third (44/137) of the Lithuanian physicians worked full-time in HF care. Among nurses, 36.2% (59/163) of Norwegian nurses worked full-time, whereas 66.5% (115/173) in Lithuania did. All participants had substantial experience with ICT, with a variance in use of programs such as Word, PowerPoint, or Excel and use of email on a mobile phone.

### Experiences and Familiarity With Noninvasive Telemonitoring in Heart Failure Care

TM is not a part of routine clinical practice in HF care in Norway or Lithuania. None of the responding HCPs were using TM. Nevertheless, a minority of HCPs in both countries confirmed to be familiar with TM in Norway (48/226, 21.2%) and in Lithuania (64/310, 20.6%).

### Relevance for Follow-Up of Heart Failure Patients Today and in Future Care

**Figure 1:**
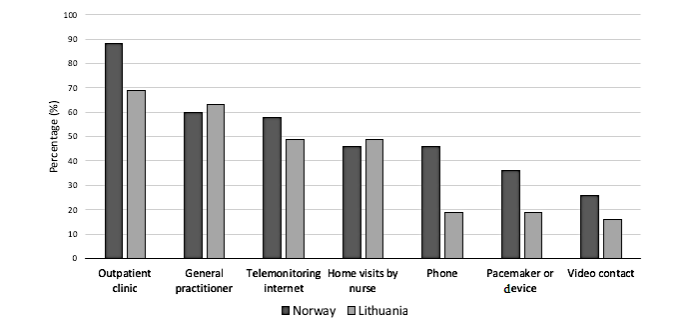
Health care practitioners’ (HCPs’) opinion of good ways of performing follow-up of stable heart failure patients. HCPs in Norway (N=226) and Lithuania (N=310). More than 1 answer was possible.

As shown in Figure 1, HCPs responded that good ways of performing follow-up of stable HF patients were by a outpatient clinic, or GP as the most optimal way to follow up HF patients. HCPs in both countries supported the potential for internet-based TM in Norway (131/226, 58.0%) and in Lithuania (152/ 310, 49.0%). A nurse-led HF outpatient clinic was specifically commented by Norwegian HCPs as an item in the column *other*, not presented in Figure 1.

Although the HCPs in Norway and in Lithuania were nonusers of TM, they considered use of TM in future HF care to be relevant. They suggested TM is useful to monitor HF patients’ physical condition signaling deterioration (in Norway: 187/226, 82.7% and in Lithuania: 225/310, 72.6%); to monitor the effect of the treatment and adjusting it remotely (140/226, 61.9% and 226/310, 72.9%); for patient education (104/226, 46.0% and 195/310, 62.9%) and remote drug titration (76/226, 33.6% and 190/310, 61.3%).

More than half of HCPs reported TM to be relevant in Norway (131/226, 58.0%) and in Lithuania (172/310, 55.5%) as shown in Table 2. The main responses to the open-ended item *other* were “I do not know,” “I lack knowledge,” and “there is a lack of evidence supporting the use of TM.”

HCPs reported that daily feedback to HF patients using TM was more feasible (115/226, 50.9%) in Norway than in Lithuania (75/310, 24.2%), whereas the “I do not know” category was chosen more frequently in Lithuania (201/310, 64.8%) than in Norway (77/226, 34.1%).

### Reasons to Consider Introducing Noninvasive Telemonitoring

The 3 most frequently mentioned reasons given by physicians and nurses in Norway and physicians in Lithuania for introducing TM to HF patients were to (1) improve patient self-care, (2) reduce hospitalizations, and (3) offer higher-quality care. The Lithuanian nurses’ 3 most frequently mentioned reasons for introducing TM to HF patients were (1) to offer higher-quality care, (2) the ability to treat more patients, and (3) to reduce the workload of the outpatient clinic as shown in Table 3.

The statement “to introduce TM for health care authorities” had the lowest score for physicians and nurses in both countries.

**Table 2:** Relevance of telemonitoring in Norway (N=226) and in Lithuania (N=310).

Relevance	Norway, n (%)	Lithuania, n (%)
Very relevant	44 (19.5)	42 (13.5)
Relevant	131 (58.0)	172 (55.5)
Not relevant	17 (7.5)	51 (16.5)
Other	24 (10.6)	33 (10.6)
Unreported or missing	10 (4.4)	12 (3.9)

**Table 3:** Physicians’ and nurses’ perception of important reasons for introducing telemonitoring into clinical practice in Norway (N=226) and Lithuania (N=310).

Reasons for introducing telemonitoring		Norway, mean score (SEM^a^)	Lithuania, mean score (SEM)
**Physicians**		**n=63**	**n=137**
	Reduce admissions or readmissions	6.85 (0.37)	8.08 (0.21)
	Improve self-care of HF^b^ patients	7.00 (0.34)	7.92 (0.20)
	Offering higher-quality care	6.50 (0.42)	8.25 (0.20)
	Ability to treat more patients	6.02 (0.40)	6.87 (0.25)
	Improve adherence to HF guidelines	6.09 (0.39)	7.75 (0.20)
	Reducing the workload on the HF outpatient clinic	5.64 (0.39)	7.80 (0.22)
	Reducing costs	5.00 (0.38)	6.45 (0.29)
	Our center is innovative	4.65 (0.38)	6.41 (0.29)
	Implementing the vision of the hospital	3.98 (0.41)	6.23 (0.29)
	Important for health authorities	3.81 (0.45)	3.58 (0.33)
**Nurses**		**n=163**	**n=173**
	Reduce admissions or readmissions	8.11 (0.18)	7.46 (0.19)
	Improve self-care of HF patients	8.03 (0.18)	7.36 (0.21)
	Offering higher-quality care	7.84 (0.21)	7.69 (0.21)
	Ability to treat more patients	7.22 (0.21)	7.62 (0.21)
	Improve adherence to HF guidelines	6.92 (0.21)	6.91 (0.20)
	Reducing the workload on the HF outpatient clinic	6.34 (0.23)	7.57 (0.19)
	Reducing costs	6.52 (0.22)	6.41 (0.27)
	Our center is innovative	6.10 (0.26)	6.42 (0.27)
	Implementing the vision of the hospital	5.31 (0.25)	6.43 (0.25)
	Important for health authorities	3.81 (0.25)	4.99 (0.30)

^a^SEM: standard error of the mean.

^b^HF: heart failure.

The criterion most often identified by all HCPs for treatment with TM was admission or readmissions in Norway (136/226, 60.2%) and Lithuania (217/310, 70.0%). HCPs in Lithuania more often mentioned New York Heart Association Functional Classification (191/310, 61.6%) than Norwegian participants (112/226, 49.6%) as a criterion for treatment with TM. The criterion patient education was supported by HCPs in Norway (116/226, 51.3%) and Lithuania (145/310, 46.8%), and the criterion adherence to medication was supported by HCPs in Norway (98/226, 43.4%) and Lithuania (169/310, 54.6%). Respondents were less interested in support and advice as a criterion for treatment with TM in Norway (93/226, 41.2%) and in Lithuania (68/310, 21.9%).

HCPs in both countries supported that TM should be used as long as necessary (Norway: 159/226, 70.4% and Lithuania: 183/310, 59.0%) or for unlimited time (Norway: 9/226, 3.9% and Lithuania: 79/310, 25.5%), with a few nonresponding in Norway (18/226, 8.0%).

### Barriers to Implementing Noninvasive Telemonitoring

HCPs reported from a list of barriers to implementing the TM in the same order: lack of financing (Norway and Lithuania, 156/226, 69.0% and 277/310, 89.4%, respectively); lack of equipment (124/226, 54.9% and 252/310, 81.3%, respectively); lack of knowledge (87/226, 38.5% and 227/310, 73.2%, respectively); and lack of guidelines from health care authorities (39/226, 17.3% and 188/310, 60.6%, respectively). The HCPs reported other barriers as shortage of staff, security issues, and need of more documentation. The HCPs’ own views of what they perceive as barriers to implement TM in both countries are shown in Table 4.

**Table 4:** Perception of health care professionals on barriers to implement telemonitoring in Norway (N=226) and Lithuania (N=310).

Barriers to implement telemonitoring			Norway, n (%)	Lithuania, n (%)
**Financing^a^**				
	**Health care authorities**			
		Regional health authorities, Ministry of health care services	123 (62.1)	87 (38.7)
		Specialist health care services, Territorial Patient Fund	25 (12.6)	85 (37.8)
		I do not know	29 (14.6)	23 (10.2)
		Unreported or missing	28 (14.1)	85 (37.8)
**Patients limitations^b^**				
	**Mental and physical limitations**			
		Mental limitations	91 (48.4)	22 (13.9)
		Acute or physical limitation	57 (30.3)	52 (32.9)
		Age limitations	48 (25.5)	6 (3.8)
	**Knowledge limitations**			
		Technical skills	44 (23.4)	14 (8.9)
		Compliance issues	14 (7.4)	11 (7.0)
		I do not know	15 (8.0)	46 (29.1)
		Unreported or missing	28 (12.4)	152 (49.0)

^a^In Norway, a total of 198 out of 226 (87.6%) participants responded to the open-ended question and a total of 225 out of 310 (72.6%) HCPs in Lithuania. More than 1 answer was possible.

^b^In Norway, a total of 188 out of 226 (83.2%) participants responded to the open-ended question and a total of 158 out of 310 (51.0%) HCPs in Lithuania. More than 1 answer was possible.

From the open-ended questions regarding funding, we found that health care authorities should be the main contributors of funding TM. Less than 5% of HCPs from both countries suggested other funding options such as from patients, the company involved, or the private sector. HCPs in Lithuania considered the European Union (EU) as a source of funding. Less than 5% of HCPs in Norway questioned whether there should be funding for TM. The barriers related to HF patients were their physical or mental condition, age, insufficient knowledge of technology, and adherence issues (eg, an acute HF condition, dementia, and cognitive or physical alterations caused by medication). Less than 5% of HCPs in both countries reported that limited access to health care services when using TM and patients with foreign language limitations were challenges to TM implementation. Access to the internet was a specific challenge mentioned by the HCPs in Lithuania.

## Discussion

### Principal Findings

Physicians and nurses working in Norwegian and Lithuanian hospitals are currently nonusers of noninvasive TM. However, HCPs in our study perceive noninvasive TM as a possibility in the future to improve the quality of care for HF patients at home. The findings of this study are in line with previously reported low use of TM in clinical practice [3,20]. In Norway, telemedicine was implemented in the 1990s; however, during a 5-year follow-up (2009-2013), the level of use was low compared with outpatient visits [33]. Moreover, Norway and Lithuania participated in a European survey that concludes that TM is not widely applied and has a potential for improving support to patients with chronic conditions in their home [34]. This is an example of health care providers lagging behind in implementing eHealth solutions as suggested by the eHealth Action Plan (2012-2020) from the European commission [35]. The eHealth position statement by the ESC [22] raises the same issues as our findings, namely, the problems with low awareness and use. HCPs’ familiarity with noninvasive TM was low in both countries (21.2% in Norway and 20.6% in Lithuania). At HF conferences, research on the use of noninvasive TM in HF care is presented, which may be a reason why a minority of HCPs in both countries were familiar with the term and what it stood for.

Although noninvasive TM is currently not in use in Norway or Lithuania, HCPs perceive TM to be relevant in future HF care, in line with the health care authorities in both countries [25,26] as well as HCPs’ expectations in Sweden and Japan [20]. All HCPs expected TM to improve the quality of care for HF patients, whereas improvements to patients’ self-care were mainly mentioned by physicians in both countries and nurses in Norway. Self-care is a cornerstone in maintaining and managing life with a chronic disease such as HF [36], and HCPs have a responsibility in educating HF patients about self-care [7,37]. Nurses in Lithuania most often mentioned treating more HF patients and reducing their workload as reasons for implementing noninvasive TM. The differences in response by nurses can be explained by the ratio of nurses in the 2 countries, with 7.7 nurses per 1000 inhabitants in Lithuania and 17.7 nurses per 1000 inhabitants in Norway [38]. Moreover, toward 2030, there is an increased need for human resources in health care globally [39] and this challenges the current preference of face-to-face follow-up of stable HF patients reported by our nurses and physicians. Furthermore, the Norwegian nurses work part-time at HF outpatient clinics, whereas physicians work full-time, for example, at hospital wards and HF outpatient clinics, which could explain why a nurse-led heart failure outpatient clinic was specifically mentioned by physicians and nurses in Norway. However, the least frequently mentioned reason for all HCPs was to implement TM because it is important for health care authorities. Therefore, it is important to consider the perceptions of nonusers of HCPs regarding the relevance and reasons for implementing noninvasive TM in future HF care in different countries.

A barrier to implementing noninvasive TM in HF clinical practice was funding and for health care authorities, as suggested by our HCPs, to be the main contributor as confirmed in previous studies [2,20,21,40]. In Norway, this is represented by national and specialized or municipal health care authorities and in Lithuania, by the Ministry of Health Care Services and the Territorial Patient Fund. A potential source to the differences in HCPs’ responses is the financial situation, with Norway a high-income country and Lithuania a middle-income country [27,28]. The EU was mentioned as a potential source for funding TM by HCPs from Lithuania, as Lithuania is a member state and has received funding for health care services from the EU [41]. HCPs in Norway questioned if TM should be funded with the lack of evidence for noninvasive TM and not being a part of current HF guidelines [7]. Moreover, the cost of implementing TM in the management of HF is not clearly reported, varying from low-to-high cost depending on how costs are measured, for example, equipment, follow-up, or hospital admission [13,23,42]. Our participants’ concern regarding shortage of staff is relevant as the workload for HCPs can increase [29,40] because nurses or physicians at hospitals, HF clinics, or telemedical centers mainly interpret the transmitted measurements [12]. Furthermore, not all the work done by nurses and physicians is visible, but the *invisible* work performed by HCPs impacts patients’ ability to manage the use of TM in their home [43]. These differences call for additional funding in high- and middle-income countries to facilitate TM implementation.

The HF patient’s condition, age, and insufficient knowledge of technology were barriers perceived by the HCPs to implementing noninvasive TM. Moreover, TM was mentioned as useful for HF patients in monitoring their HF signaling a worsening condition and reducing hospitalization. Our participants most often mentioned the criterion of readmission or admission to hospital when providing examples of participants eligible for TM. More recent noninvasive TM research with HF patients shows potential to reduce the risk of hospitalization [44] and identifies eligible HF patients [45]. Elderly HF patients without ICT competency can use new technology, and they describe better contact with HCPs [46]; however, this is not part of our findings. The lack of familiarity and high ICT competency among HCPs in our survey may be a reason why they perceived that elderly HF patients are not eligible for noninvasive TM. In Lithuania, HCPs presented a lack of internet access as a specific barrier, which may be a reason for their limitations in noninvasive TM, as the use of internet is essential in our TM definition [20,29]. To involve HCPs in an earlier phase of developing new technology is a way of making their contributions visible and acknowledged and may contribute to our participants finding noninvasive TM relevant for HF patients in Norway and Lithuania.

### Strengths and Limitations

The fairly high response rate is a strength of the study. HCPs from both hospital wards, which discharge patients to their homes, and outpatient clinics, which see patients who live at home, were asked to participate. There are several limitations to our study. First, the self-reported questionnaire does not provide in-depth knowledge about HCPs’ knowledge of TM. Second, it was not possible to sample HCPs’ experiences with TM in HF clinical practice as none of the hospitals were using TM at the time of the survey. This was not anticipated. This shortfall implies that some questionnaire items need to be revised. Furthermore, a comprehensive assessment of the questionnaire’s face and content validity may have strengthened the study.

### Conclusions

HCPs in Norway and in Lithuania are currently nonusers of TM in daily follow-up of HF care; however, they perceive that a future use of TM is relevant to improve the quality of care for HF patients. From the perspective of physicians in both countries and Norwegian nurses, the main reason for introducing noninvasive TM in HF care was to improve patient’s self-care. The nurses in Lithuania expected to treat more HF patients and reduce their workload by implementing TM. Financial barriers and HF patients’ condition may have an impact on the use of TM, whereas sufficient funding from health care authorities and improved knowledge may encourage the more widespread use of TM in the Nordic Baltic region and beyond.

## Appendix

Multimedia Appendix 1 Questionnaire used in this survey.

## Abbreviations

eHealth: electronic health
ESC: European Society of Cardiology
EU: European Union
GP: general practitioner
HCP: health care professional
HF: heart failure
ICT: information and communications technology
TM: telemonitoring
