# Supplementary material for: Health Care Professionals’ Perceptions of Home Telemonitoring in Heart Failure Care: Cross-Sectional Survey
Source: J Med Internet Res. 2019 Feb 6;21(2):e10362. doi: 10.2196/10362 (PMC6381407; doi:10.2196/10362)
Supplement: Multimedia Appendix 1 [file jmir_v21i2e10362_app1.pdf]

Perceptions of and experiences with telemonitoring in the management of patients with heart failure

This questionnaire concerns your opinion about and experiences with telemonitoring when caring for patients with heart failure

DEFINITION OF TELEMONITORING

In this Questionnaire telemonitoring **refers to non-invasive remote, Internet-based monitoring body weight, blood pressure, heart rate, dyspnea and other signs and symptoms** that would reflect the actual volume status of heart failure patients. The devices are used by the patients in their own home environment and the generated data are transferred to health care providers over the Internet. The information obtained is presumed to disclose the actual condition of heart failure patients..
The use of telemonitoring by means of **only telephone, telephone support, telephone follow-up, or by means of implantable devices/pacemakers is not included in this study.**

Filling in the questionnaire will take about 10 minutes.

The questionnaire consists of three parts. All participants fill out part 1 and part 2 or part 3.
Part 1. General questions and Telemonitoring for all to answer.
Part 2. User questions. Only for users of telemonitoring.
Part 3. Non-Users questions. Only for non-users of telemonitoring.

PLEASE FILL IN OR MARK THE ANSWER THAT BEST APPLIES TO YOU FOR THE GENERAL QUESTIONS LISTED BELOW.

1 What is your gender?

☐ Male ☐ Female

2 How old are you?

☐ Years

3 What is your highest educational degree?

☐ Doctoral education

☐ Master

☐ Bachelors

☐ None

☐ Other type of degree, namely:.......................

4 What is your current job?

☐ Physician/Cardiologist / Physician specializing in cardiology

☐ Nurse/Specialist Nurse

☐ Other, namely:...

5 How many years of postgraduate experience do you have?

☐ Years

6 Do you work full time or part time with heart failure patients?

☐ Full time

☐ Part time......Days

☐ Part time....Hours

7a What is the name of your hospital/institution?

....

7b What kind of hospital is it?

☐ University hospital

☐ Secondary level hospital

☐ Third level hospital

☐ Private hospital

☐ Other, namely:...

8 **How many years of computer experience do you have (both work and private)?**

☐ Years

9 **Do you have experience with operating systems such as Windows or Apple?**

☐ Yes ☐ No

10 **Do you have experience with programmes such as Word, PowerPoint, Excel or ITunes?**

☐ Yes ☐ No

11 **Do you have experience with programming languages, e.g. Pascal, Java, C++?**

☐ Yes ☐ No

12a **Do you know how to use email?**

☐ Yes ☐ No

12b **Do you have experience with using e-mail on your mobile phone?**

☐ Yes ☐ No

13 **Do you have experience with using the internet?**

☐ Yes ☐ No

14 **Which according to you would be good ways of performing follow-up of stable HF patients? (more than one answer is possible)**

- ☐ By telemonitoring using internet-based monitoring
- ☐ By making use of pacemaker/ device based data
- ☐ By phone
- ☐ By video contact (for ex. Skype)
- ☐ By e-mail on mobile phone
- ☐ Outpatient clinics
- ☐ By General practitioner
- ☐ Home visits by nurse
- ☐ Home visit by other person
- ☐ Other, namely:..

15 **Are you familiar with heart failure telemonitoring?**

☐ Yes ☐ No

TELEMONITORING

16 **How easy/feasible do you think it is to give daily feedback to patients via telemonitoring?**

- ☐ Very feasible/easy
- ☐ Feasible/easy
- ☐ Not feasible/easy
- ☐ I don't know

17 **Who should be responsible for the funding of telemonitoring?**

....

....

18 **In which situation would you believe that telemonitoring is not appropriate?**

....

....

19 **How relevant is Telemonitoring in Norway?**

- ☐ Very relevant
- ☐ Relevant
- ☐ Not relevant
- ☐ Other, namely:..

20 **What do you consider as the most relevant technical development that can be used NOW in heart failure care?**

....

....

....

....

21 **What do you consider as the most relevant technical development that can be used in the FUTURE in heart failure care?**

....

....

....

22 Do you have knowledge of heart failure clinics in Norway that use telemonitoring?

☐ Yes, namely ....

☐ No

23 Does your heart failure clinic/cardiology clinic use telemonitoring?

☐ Yes → Please go to Users Questions (Page ??)

☐ No → Please go to Non-users Questions (Page ??)

USERS QUESTIONS

24 Please indicate on a scale between 0 and 10 how important the following reasons were in introducing telemonitoring to your centre?

0 = completely unimportant - 10 = very important

Importance of introducing telemonitoring	Mark 0 - 10
1. Offering higher-quality care	
2. Reducing costs	
3. Implementing the vision/goal of the hospital	
4. Ability to treat more patients	
5. Reducing the workload on the heart failure out-patient clinic	
6. Reduce admissions/readmissions	
7. Better adherence to heart failure guidelines	
8. Improve self-care of heart failure patients	
9. Our centre is innovative	
10. It`s mainly our Health authorities thinks that it's important	

25 What is the purpose of telemonitoring your heart failure patients?

(more than one answer is possible)

☐ Monitoring physical condition / noticing a decline

☐ Monitoring the effect of the treatment and adjusting it remotely

☐ Remote drug titration

☐ Patient education

☐ Other, namely:..

26 How long is the average period for telemonitoring in your clinic?

☐ More than 3 months but no more than 6 months

☐ More than 6 months but no more than 1 year

☐ Unlimited

☐ As long as it is necessary

27 Indicate the criteria you think one should use to select patients for treatment with telemonitoring

(more than one answer is possible)

☐ NYHA class

☐ Admission or readmission

☐ Compliance of medication

☐ Patient education

☐ Support and advice

☐ Other, namely:..

28 Which telemonitoring system does your heart failure clinic use?

System/Company:..

29 Have you heard of other systems than the one you are using now?

☐ Yes, namely: ....

☐ No

30 Which system would you choose if you had the chance to or replace you current system with?

System/Company: ....

....

31 **Please indicate your opinion on a scale between 0 and 10 on the difference you experience between HF care without telemonitoring compared to HF care with telemonitoring**

0 = no difference - 10 = very large difference

Difference experienced after introduction of telemonitoring	Mark 0 - 10
1. Higher quality of care	
2. Reducing costs	
3. Increased number of treated patients	
4. Reduced workload on the heart failure out-patient clinic	
5. The hospitals goal is implemented	
6. Fewer admissions/readmissions	
7. Better adherence to heart failure guidelines	
8. Improve self-care of heart failure patients	
9. Higher degree of innovation	

32 **How many patients are currently being cared for with telemonitoring?**

- ☐ None
- ☐ 1 to 10 patients
- ☐ 11 to 20 patients
- ☐ 21 to 50 patients
- ☐ 51 to 75 patients
- ☐ More than 75 patients

33 **How many of your heart failure patients use telemonitoring?**

☐

34 **Please estimate the NYHA class of the majority of the patients currently followed with telemonitoring**

- ☐ NYHA class I
- ☐ NYHA class II
- ☐ NYHA class III
- ☐ NYHA class IV

35 **Who is responsible for the data that is generated by telemonitoring?**

....

....

36 **Do you have a maximum period from data coming in and healthcare provider responding to the patient?**

- ☐ Yes, days ....
- ☐ Yes, hours ....
- ☐ No

37 **How satisfied are you with telemonitoring so far?**

- ☐ Very dissatisfied
- ☐ Dissatisfied
- ☐ Neutral
- ☐ Satisfied
- ☐ Very satisfied

THIS IS THE END OF THE QUESTIONNAIRE, THANK YOU!

NON-USERS QUESTIONS

38 Please indicate your opinion on a scale between 0 and 10 how important the following reasons might be to introduce telemonitoring to your centre in the future?
0 = completely unimportant - 10 = very important

Importance of introducing telemonitoring	Mark 0 - 10
1. Offering higher-quality care	
2. Reducing costs	
3. Implementing the vision/goal of the hospital	
4. Ability to treat more patients	
5. Reducing the workload on the heart failure out-patient clinic	
6. Reduce admissions/readmissions	
7. Better adherence to heart failure guidelines	
8. Improve self-care of heart failure patients	
9. Our centre is innovative	
10. It`s mainly our Health authorities thinks that it's important	

39 What, in the future, do you think might be a purpose for telemonitoring of your heart failure patients?
(more than one answer is possible)

☐ Monitoring physical condition/signalling deterioration
☐ Monitoring the effect of the treatment and adjusting it remotely
☐ Remote drug titration
☐ Patient education
☐ Other, namely:...

40 How long do you think the average period for telemonitoring should be?

☐ More than 3 months but no more than 6 months
☐ More than 6 months but no more than 1 year
☐ Unlimited
☐ As long as it is necessary

41 Indicate the criteria you think one should use to select patients for treatment with telemonitoring
(more than one answer is possible)

☐ NYHA class
☐ Admission or readmission
☐ Compliance of medication
☐ Patient education
☐ Support and advice
☐ Other, namely:

42 What are the important barriers to use telemonitoring in your institution?

....

....

43 What do you need to implement telemonitoring?
(more than one answer is possible)

☐ Financing
☐ Knowledge
☐ Equipment
☐ Guidelines by health authorities
☐ Other, namely:...

This questionnaire was adapted from previous work
(de Vries et al, J Med Internet Res 2013;15(1):e4 and Kato et al www.jmir.org/2015/11/e258/)
